# Virulence difference of five type I dengue viruses and the intrinsic molecular mechanism

**DOI:** 10.1371/journal.pntd.0007202

**Published:** 2019-03-04

**Authors:** Chunling Zou, Chenxiao Huang, Jinyu Zhang, Qihan Wu, Xiaohua Ni, Jiufeng Sun, Jianfeng Dai

**Affiliations:** 1 Institutes of Biology and Medical Sciences, Jiangsu Key Laboratory of Infection and Immunity, Soochow University, Suzhou, P. R. China; 2 NHC Key Lab of Reproduction Regulation (Shanghai Institute of Planned Parenthood Research), Fudan University, Shanghai, P.R. China; 3 Guangdong Provincial Institute of Public Health, Guangdong Provincial Center for Disease Control and Prevention, Guangzhou, P.R. China; Instituto de Ciências Biológicas, Universidade Federal de Minas Gerais, BRAZIL

## Abstract

Dengue virus (DENV) is the most important vector-borne virus globally. The safe and effective vaccines are still under development and there are no antiviral drugs for DENV induced diseases. In this study, we obtained five DENV1 isolates (DENV1 A to E) from the outbreak of dengue fever in 2014 of Guangzhou, China, and analyzed their replication efficiency and virulence *in vitro* and *in vivo*. The results suggested that among the five DENV1 strains, DENV1 B has the highest replication efficiency in both human and mosquito cells *in vitro*, also causes the highest mortality to suckling mice. Further study suggested that nonstructural proteins from DENV1B have higher capacity to suppress host interferon signaling. In addition, the NS2B3 protease from DENV1B has higher enzymatic activity compared with that from DENV1 E. Finally, we identified that the 64^th^ amino acid of NS2A and the 55^th^ amino acid of NS2B were two virulence determining sites for DENV1. This study provided new evidences of the molecular mechanisms of DENV virulence.

## Author summary

Dengue is the most important vector-borne viral infection that endangers an estimated 2.5 billion people globally. The recently licensed dengue vaccine has major weaknesses and there are no antiviral drugs for the treatment of dengue related diseases. Identifying the virulence determinants is important for understanding the molecule bases of viral life cycle, also contributing to vaccine design and development. In this study, we analyzed the virulence differences among five DENV1 strains that obtained from the 2014 DENV outbreak in Guangzhou, China, and identified two novel virulence determining sites for DENV1. This study provides new ideas for investigation of DENV protein function, pathogenic mechanism and novel attenuated vaccine.

## Introduction

Dengue virus (DENV) is currently the most popular mosquito-borne virus and widely spreads in tropical and subtropical regions[1]. A series of symptoms caused by DENV, such as dengue hemorrhagic fever/dengue shock syndrome (DHF/DSS), seriously threaten human health[2,3]. Dengue virus belongs to a single-stranded positive sense RNA virus and lacks of accurate replication correcting system. Virus nucleotide changes will eventually lead to stronger or weaker virus virulence during the long-term process of virus spread. Secondly, host response is induced upon virus infection and the interactions between host and virus also influence the virulence. These two aspects corporately influence the virus pathogenicity and severity of the diseases[4,5]. Therefore, it is of great significance to identify the sites within the virus genome that are associated with virulence and to investigate the interactions between virus and host.

DENV genome is an approximately 10.7-kb positive-sense RNA, encodes a single polyprotein that is cleaved posttranslationally by host and viral proteases into three structural proteins (capsid [C], premembrane [prM], and envelope [E]) and seven nonstructural proteins (NS1, NS2A, NS2B, NS3, NS4A, NS4B, and NS5). Structure proteins C, M and E are components of viral particles, and the viral nonstructural proteins are critical for viral genome transcription and replication[5]. At the same time, flavivirus nonstructural proteins are reported to attenuate host antiviral responses and facilitate viral survival[6]. For example, Dengue virus (DENV) NS4B and NS4A inhibited TBK1 phosphorylation thereby reducing the IFN production[7]. DENV NS2A, 2B, 4B and NS5 inhibit IFN-mediated JAK-STAT activation and impair interferon-stimulated gene (ISG) production[8–12]. DENV NS5 could inhibit STAT2 phosphorylation thereby blocking IFN downstream signaling[13]. The amino acid changes in the nonstructural proteins were believed to influence the activity of virus to antagonize host antiviral responses.

The variations in virus genome are closely related with the virulence. Lots of studies showed that the mutations in virus E protein resulted in the significant changes in virulence of flavivirus[14]. By using the reverse genetic approaches, Prestwood and collaborators found mutations at amino acid 124 and 128 in E protein increased the virulence of DENV[15]. Three substitutions in E protein (196Met→Val, 365Val→Ile,405Thr→Ile) and one in NS3 protein (435Leu→Ser) were reported to be associated with the pathogenesis of neurotoxicity of DENV[16,17]. As a multiple function protein, NS3 has serine protease activity in its N terminal domain, which is required for the cleavage of viral polyprotein and the maturation of viral particles. The NS3 protease cleavage ability will determine the assembling efficiency of viral particles[18,19]. Furthermore, the hypervariable regions of the 3’UTR are also believed to be the virulence determination sites, although the biological relevance is remained to be elucidated[20,21].

In the past 25 years, cases of dengue infection have been reported in Guangdong province of China every year[22–24]. The peak of infection happened in 2014, and DENV1 appeared as the major serotype at that year[23,25]. We acquired five DENV1 isolates with different genotypes from Guangdong province among the outbreak of dengue fever of 2014. The differences between these virus strains were studied in *in vitro* and *in vivo* models. To further understand the molecular mechanism underlying the virulence difference of five variants, we investigated the ability of the variants antagonizing host innate immune response and the functions of their NS2B3 protease.

## Results

### The virulence difference among five DENV1 strains

Five different DENV1 isolates, named DENV1A to DENV1E in this study, were isolated from the DENV outbreak of 2014 in Guangdong Province, China. The nucleotide sequences of these five DENV1 strains were determined by high throughput sequencing/assembling approach and submitted to Genebank under the accession numbers MH271402 (DENV1A) to MH271406 (DENV1E).

**Fig 1:**
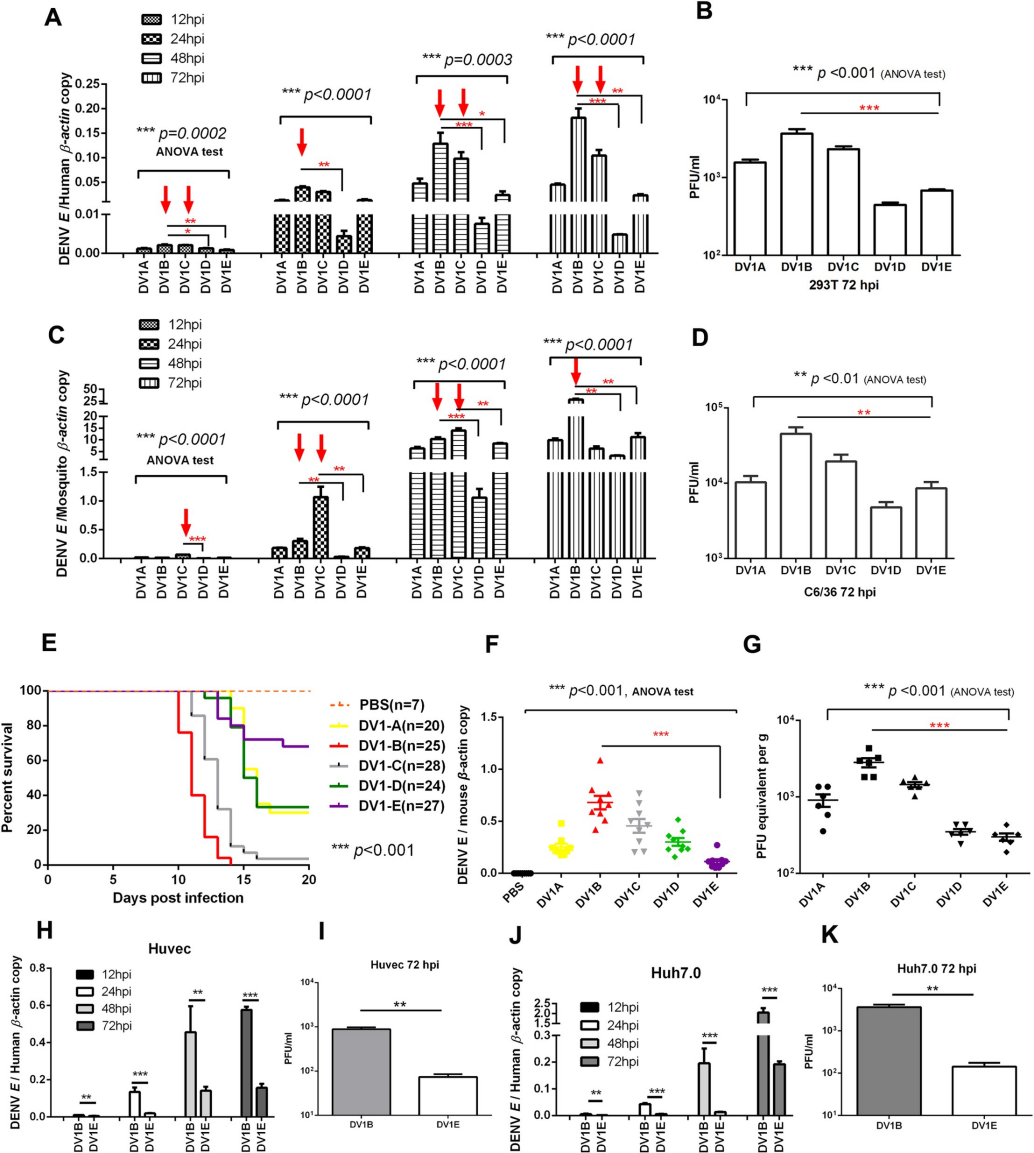
The virulence difference among five DENV1 strains. (A) The replication of DENV1A-E (labeled as short form of DV1A to DV1D in the figures) in human in 293T cells. 293T cells were infected with DENV1 A to E at an MOI of 0.5, respectively. Cells were harvested at 12, 24, 48 and 72 h post infection, and viral replication efficiency were determined by analyzing viral E gene mRNA copies and normalized to human *β-actin* gene. (Results were shown as Mean±SEM, **p<0.01, ***p<0.001, ANOVA test, **p<0.01, ***p<0.001, Tukey’s multiple tests. The red arrows indicated the significantly high replications of isolates) (B) The viral titers in supernatants of 293T cells at 72h post infection. Virus titration was performed with a modified TCID_50_/plaque assay as described in the Method section. (C) The replication of DENV1 A-E in mosquito C6/36 cells. Viral mRNA copies were normalized to mosquito *β-actin* gene. (D) The viral titers in supernatants of C6/36 cells at 72h post infection. (E) The survival of DENV1 A-E infected suckling mice. (***p<0.001, Log-rank test). (F) Viral loads in brain tissues from DENV1 A-E infected suckling mice. (G) The virus titers in the tissue extracts from brains of infected suckling mice. (Results were shown as Mean±SEM, *** *p*<0.001, ANOVA test, ***p<0.001, Tukey’s multiple tests.) (H-I) The viral RNA replication (H) and virus production (I) of DENV1 B and E in HUVEC cells. (J-K) The viral RNA replication (J) and virus production (K) of DENV1 B and E in Huh7.0 cells. (H-K: results were shown as Mean±SEM, **p<0.01, ***p<0.001, *t*- test). The data shown are representative of at least 3 independent experiments.

To test the replication efficiency of these five isolates in mammalian cells, human 293T cells were infected with DENV1 A to E respectively at the same MOI of 0.5. Cells were harvested at 12, 24, 48 and 72 h post infections, and the viral replication efficiency were determined by measuring viral envelope (E) gene mRNA copies, then normalized to human *β*-actin gene. The results suggested that DENV1B and DENV1C had higher replication efficiency among the five virus strains in 293T cells (**Fig 1A**). Consistent with the intracellular viral RNA levels, the virus titers in supernatants from DENV1B and 1C infected cells were higher than those from DENV1D and 1E infected cells (**Fig 1B**).

To test the infection ability of these five strains to mosquito cells, C6/36 cells (origin from *Aedes albopictus*) were infected with DENV1 A to E. The results suggested that DENV1B also showed higher replication efficiency and produced more viral particles in mosquito cells when compared with other strains (**Fig 1C and 1D**).

Then we used a sucking mice infection model to test the virulence of these five DENV1 strains *in vivo*. Three- or four-day-old suckling mice were inoculated intracerebrally with 100 PFU of DENV1 A to E respectively, and the mortality rates were monitored daily. The results suggested that sucking mice started to die at 10 days post DENV1B infection, while this date was postponed to day 15 in case of DENV1E infection. 100% of DENV1B infected mice died at day 15 post infection, but 70% of DENV1E infected mice were survived from infection and recovered (**Fig 1E**). These data suggested that DENV1B is the most virulent virus, while DENV1E is the weakest. We also quantified the viral loads in infected mouse brains by qRT-PCR and plaque assays. Consistently, the viral loads in the brains of DENV1B infected mice were significantly higher than those from DENV1E infected mice (**Fig 1F and 1G**).

To further confirm that DENV1B is the most virulent virus while the DENV1E is the weakest, HUVEC and Huh7.0 cells were infected with DENV1B and E, respectively. The results suggested that DENV1B showed significantly higher replication levels at all time points post infection and produced more viral particles in these cells (**Fig 1H to 1K**). Taken together, these data suggested that DENV1B is the most virulent virus among the five DENV1 strains, while DENV1E is the weakest.

### Molecular evolution analysis of five DENV1 strains

**Fig 2:**
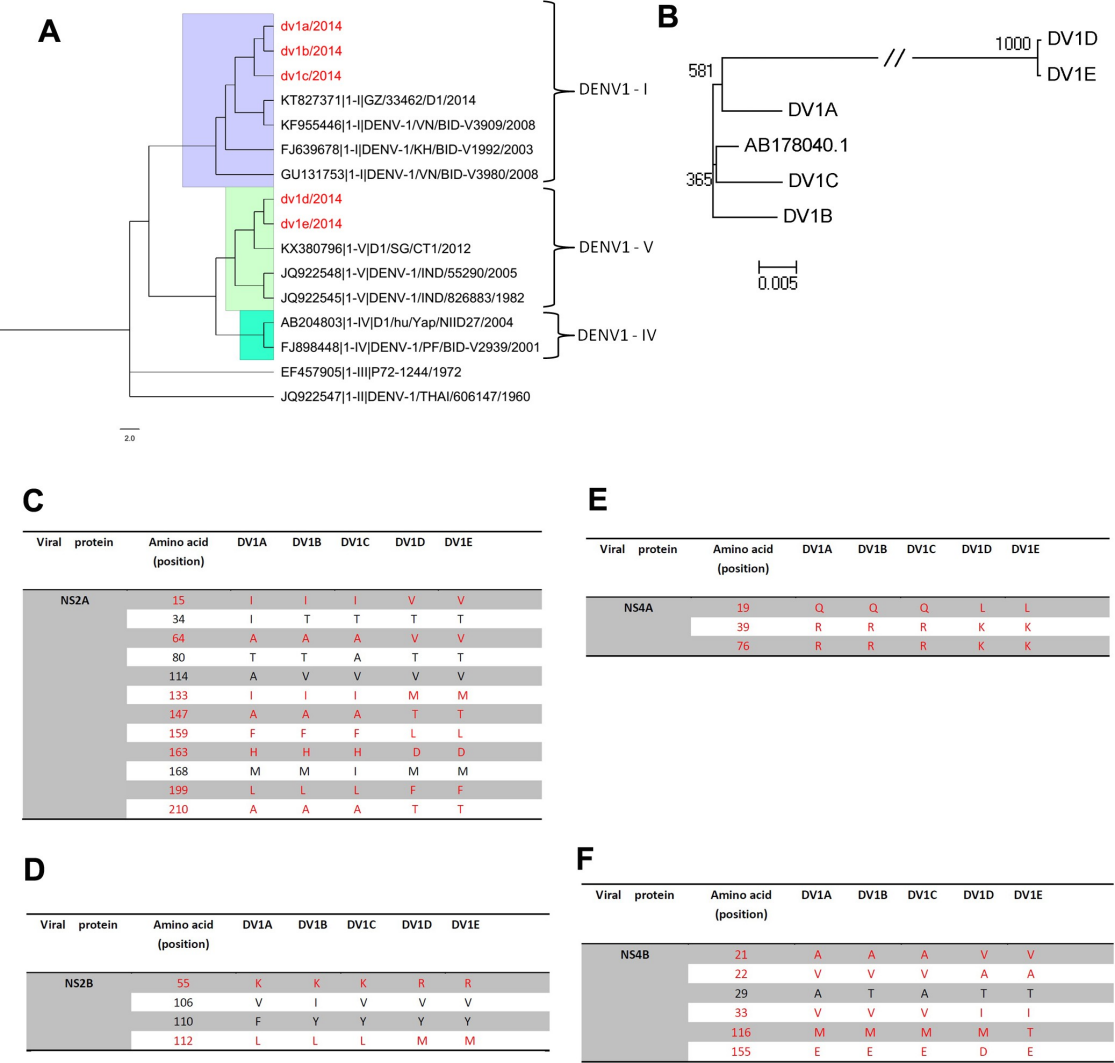
Molecular evolution analysis of five DENV1 strains. (A) Phylogenetic tree of DENV1A-E and other 11 representative DENV1 viruses. DENV1 genotype cluster I, IV and V were indicated. (B) Phylogenetic tree of DENV1A-E and the DENV1 strain used for the construction of DENV1 replicon DGL2 (AB178040.1). (C-F) The amino acid variations of NS2A (C), 2B (D), 4A (E) and 4B (F) from DENV1 A to E.

DENV-1 can be divided into 5 main genotypes. Genotype II and III have only a few early strains, and I, IV and V are the three major genotypes in circulation[26,27]. In order to understand the origin and relationships of the five DENV-1 viruses, molecular evolution analysis was performed using various bioinformatics approaches. 123 full length DENV1 sequences were downloaded from ViPR (www.viprbrc.org) database. The ORFs of these 123 viruses, together with DENV1 A to E from this study, were analyzed by Multiple alignment program Mafft (https://mafft.cbrc.jp/alignment/server/). The phylogenetic tree showed that DENV1A, B and C belong to genotype I of DENV1 (**Fig 2A, and S1 Fig**). DENV1A is closely related with the DENV1 viruses that are endemic in Zhongshan (China) (2013) and Shizuoka (Japan) (2014). DENV1 B and C are closely related to LC011948, which is endemic in Chiba, Japan in 2014. Then we speculated that the strains erupted in Guangdong and Japan in 2013–2015 probably came from the same ancestor. DENV1D and 1E are very similar to each other. They are located in a branch of DENV1-V genotype, share homology with DENV-1 (KX380801 and KX380796) isolated from Singapore in 2014 (**Fig 2A, and S1 Fig**). In addition, DENV1B and C share high similarities to a DENV1 strain (AB178040.1) isolated from Japan in 2004 (**Fig 2B**). Then, a DENV1 replicon, DGL2, origin from this DENV1 strain[28] was used as a reverse genetics approach to perform the single point mutations in DENV1B genomes.

Using MEGA software, the ancestral sequences for the five DENV1 viruses were analyzed and 72 amino acid variations between DENV1 A&B&C and DENV1 D&E were identified (**S2 Table**). At the same time, all the variations in NS2A, 2B, 4A and 4B among DENV1 A to E were showed in **Fig 2C–2F**.

### Antagonizing of IFN signaling by NS proteins from five DENV1 strains

**Fig 3:**
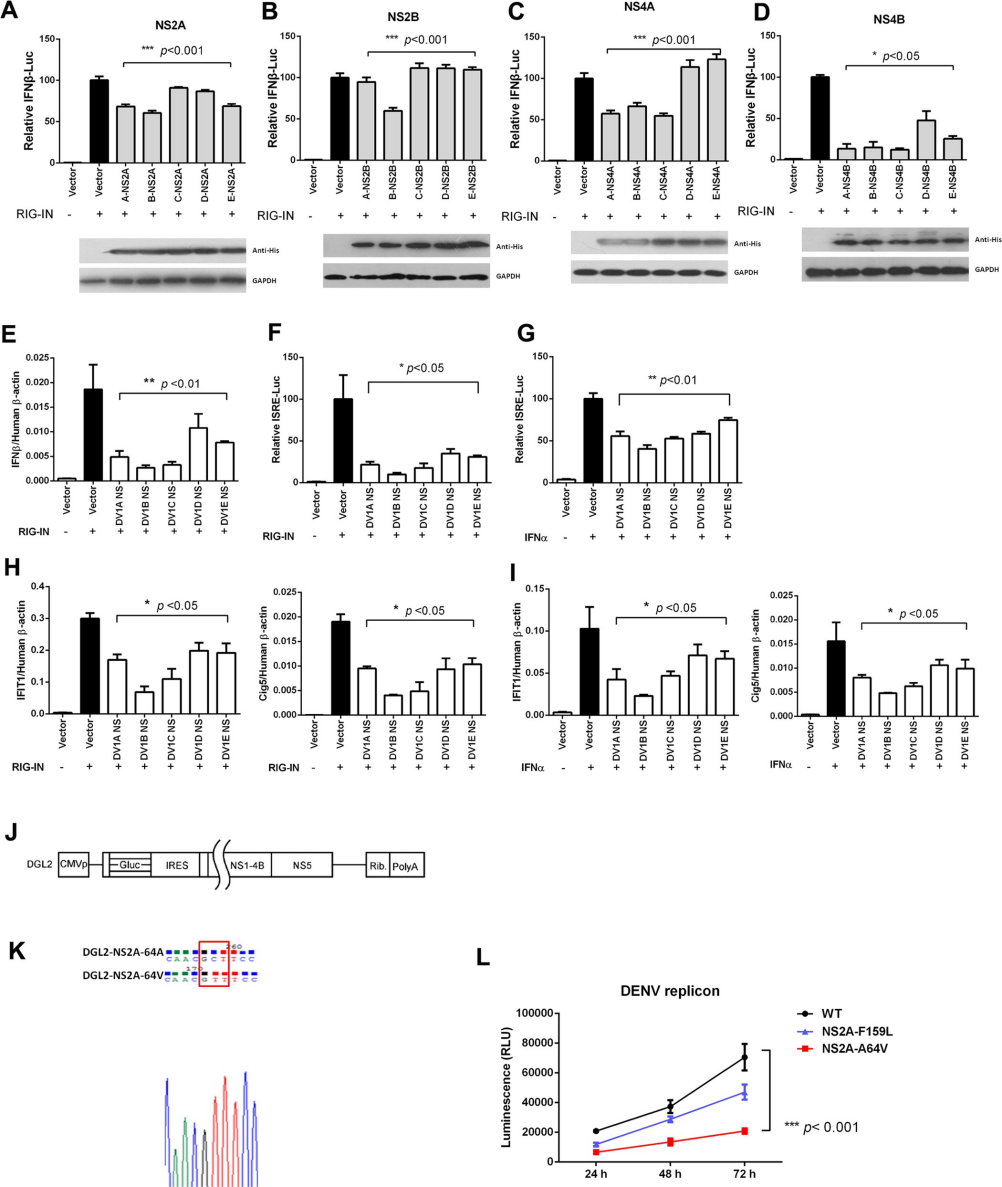
Antagonizing of IFN signaling by NS proteins from five DENV1 strains. (A-D) Overexpression of NS2A (A), 2B (B), 4A (C) and 4B (D) from DENV1 A-E suppressed RIG-I directed IFNβ-promoter activation. 293T cells were transfected with RIG-I-N, IFNβ-Luciferase reporter, together with plasmids encoding NS proteins or vector control. IFNβ-promoter activations were determined by Dual luciferase assay. (The mean values of the activated control groups were normalized as “100”). The expressions of indicated NS proteins were shown by Western Blots in the lower panels. (E) Co-expressing of four NS proteins (2A+2B+4A+4B) from DENV1A to E suppressed *IFNβ* mRNA production in RIG-I-N transfected 293T cells. (F-G) Co-expressing NS proteins (2A+2B+4A+4B) from DENV1A to E suppressed RIG-I-N (F) or IFNα (G) induced ISRE promoter activation. (H-I) Co-expressing NS proteins from DENV1A to E impaired ISG mRNA production from RIG-I-N (F) or IFNα (G) stimulated 293T cells. The relative mRNA expression levels of typical ISG genes *IFIT1* and *Cig5* were determined by qRT-PCRs. (A-I, Results were shown as Mean±SEM, * *p*<0.05, **p<0.01, ***p<0.001, ANOVA test). (J) A brief diagram of the structure of a DENV1 replicon DGL2. (K) Sequencing confirmation of a single point mutation in DGL2 replicon at position of NS2A A64V. (L) The replication of WT and mutant DGL2 replicons in 293T cells at indicated time points. (Results were shown as Mean±SEM, ***p<0.001, ANOVA test) The data shown are representative of at least 3 independent experiments.

DENV NS proteins were reported to have the ability to suppress IFN signaling, and this activity will contribute to its virulence in mammalian host. To test whether NS proteins from DENV1 A-E have different capacity against IFN signaling, NS2A, 2B, 4A and 4B from all these five strains were cloned and expressed in 293T cells. IFN-β-Luciferase reporter assay suggested that NS2A and 2B from DENV1B showed the highest inhibitory activity against RIG-I directed IFN activation, compared with NS2A/2B from other strains. NS4A and NS4B from DENV1 A&B&C also have higher inhibitory capacity to IFN signaling compared to those from DENV1 D&E (**Fig 3A–3D**). Consistent with these results, RIG-I induced *IFNβ* mRNA expression was also dramatically decreased in cells expressing NS proteins from DENV1B when compared with those from other strains (**Fig 3E**). Type one IFNs binds to interferon receptor and activates the transcription of genes containing an ISRE responsive element in their promoters. We also tested whether NS proteins from different DENV1 strains showed variable capacities to modulate the ISRE activation. The results suggested that NS proteins from DENV1B also showed the highest inhibitory activity on ISRE-Luc activity during RIG-I-N or IFNα stimulation (**Fig 3F and 3G**). Consistently, RIG-I-N or IFNα induced transcriptions of typical ISG genes, such as IFIT1 and Cig5, were significantly inhibited in DENV1B NS protein expressing cells (**Fig 3H and 3I**). In line with this, we also confirmed that DENV1B showed a better replication than DENV1E in 293T cells if we treated the cells with IFNα.

To confirm the functions of DENV1B NS2A, we constructed two mutant DENV1 replicon plasmids (NS2A A64V and F159L) based on the DGL2 replicon (**Fig 3J**) (The NS2A proteins of DGL2 are 100% identical with that of DENV1B). The 64^th^ Ala (A) and 159^th^ Phe (F) of NS2A from DENV1B were changed to Val (V) and Leu (L) (from DENV1E), respectively. DNA sequencing results indicated that the point mutation was successfully introduced into DGL2 replicon (**Fig 3K**). After transfecting these replicons into 293T cells, we found that NS2A A64V mutation significantly impaired the replication efficiency of DENV1 replicon, but F159L substitution only slightly influenced the replication (**Fig 3L**). These data suggested that NS2A amino acid position 64 is one of the important virulence determinants for DENV1.

### DENV1B shows higher replication efficiency in IFNAR1^-/-^ mice and cells

**Fig 4:**
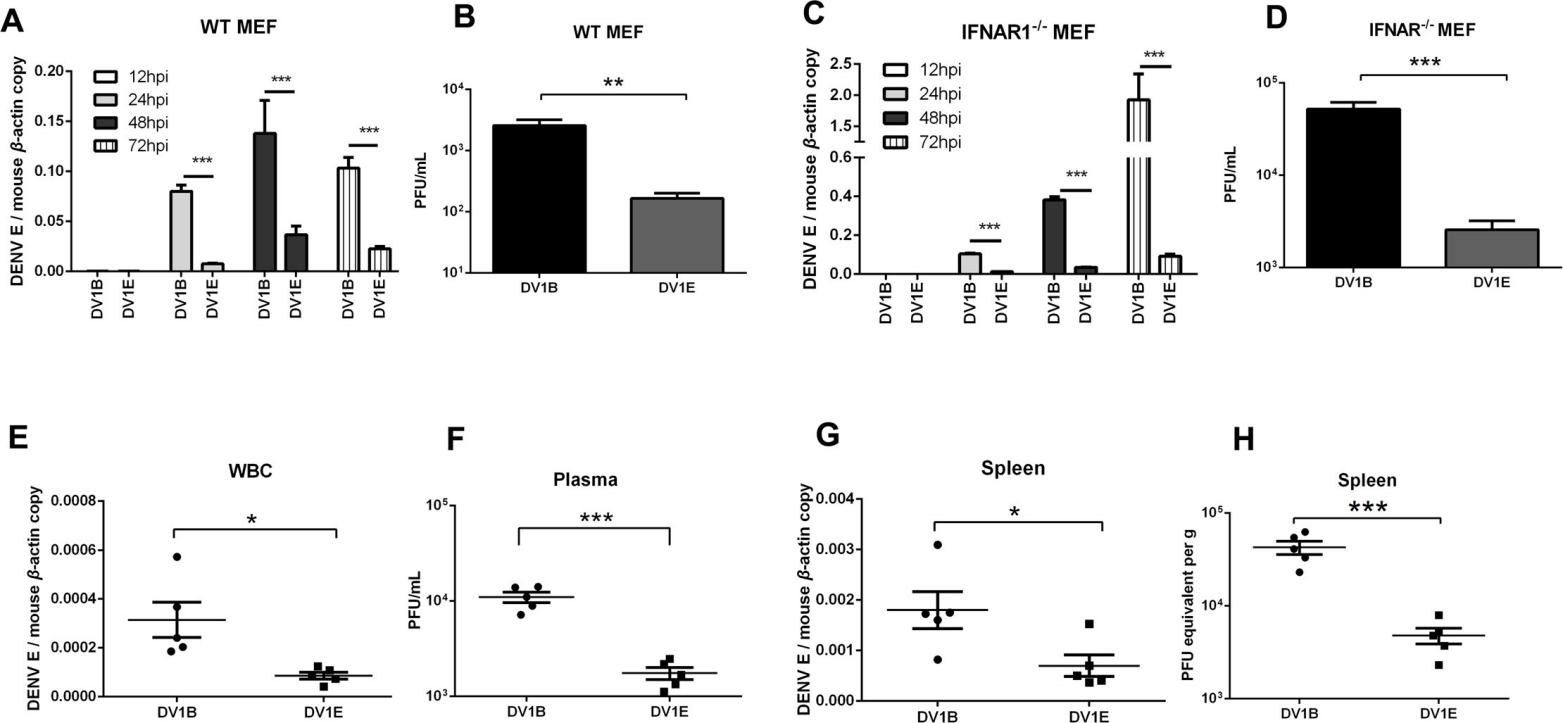
DENV1B shows higher replication efficiency in IFNAR1^-/-^ mice and cells. (A-B) The viral RNA replication (A) and virus production (B) of DENV1 B and E in in MEF cells from WT mice. (C-D) The viral RNA replication (C) and virus production (D) of DENV1 B and E in MEF cells from IFNAR1^-/-^ mice. (E-H) The viral loads in whole blood cells (WBC) (E-F) and spleens (G-H) from DENV1 B and DENV1 E infected IFNAR1^-/-^ mice. (The viral loads were determined by qRT-PCR analysis of viral genes (E and G) or plaque titration of viral particles (F and H) as described above.) Results were shown as Mean±SEM, * *p*<0.05, **p<0.01, ***p<0.001 *t*- test. The data shown are representative of at least 3 independent experiments.

Since NS proteins from DENV1B have higher capacity to inhibit IFN signaling, we wondered whether this is a major factor that determining the virulence of these five DENV1 strains. IFNAR1^-/-^ mice were introduced to study the replication efficiency of DENV1 B and E in IFN non-responsive system. MEF cells from wild type and IFNAR1^-/-^ mice were obtained and infected with DENV1 B and E, respectively. Surprisingly, DENV1B still has higher infection efficiency than DENV1E in IFNAR1^-/-^ MEFs, just like what it does in wild type MEFs (**Fig 4A–4D**). To further confirm this, IFNAR1^-/-^ mice were challenged with DENV1B and E *via* intraperitoneal infection for 3 days, then the viral loads in blood and spleens were tested by qRT-PCR and plaque assay. The results suggested that the viral load in blood and spleen samples from DENV1B infected IFNAR1^-/-^ deficient mice were significantly higher than that from DENV1E infected mice (**Fig 4E–4H**). These data suggested that the difference in antagonizing IFN signaling is not the only determination factor for the virulence of these DENV1 strains.

### NS2B K55R is a virulent determinant for DENV1

**Fig 5:**
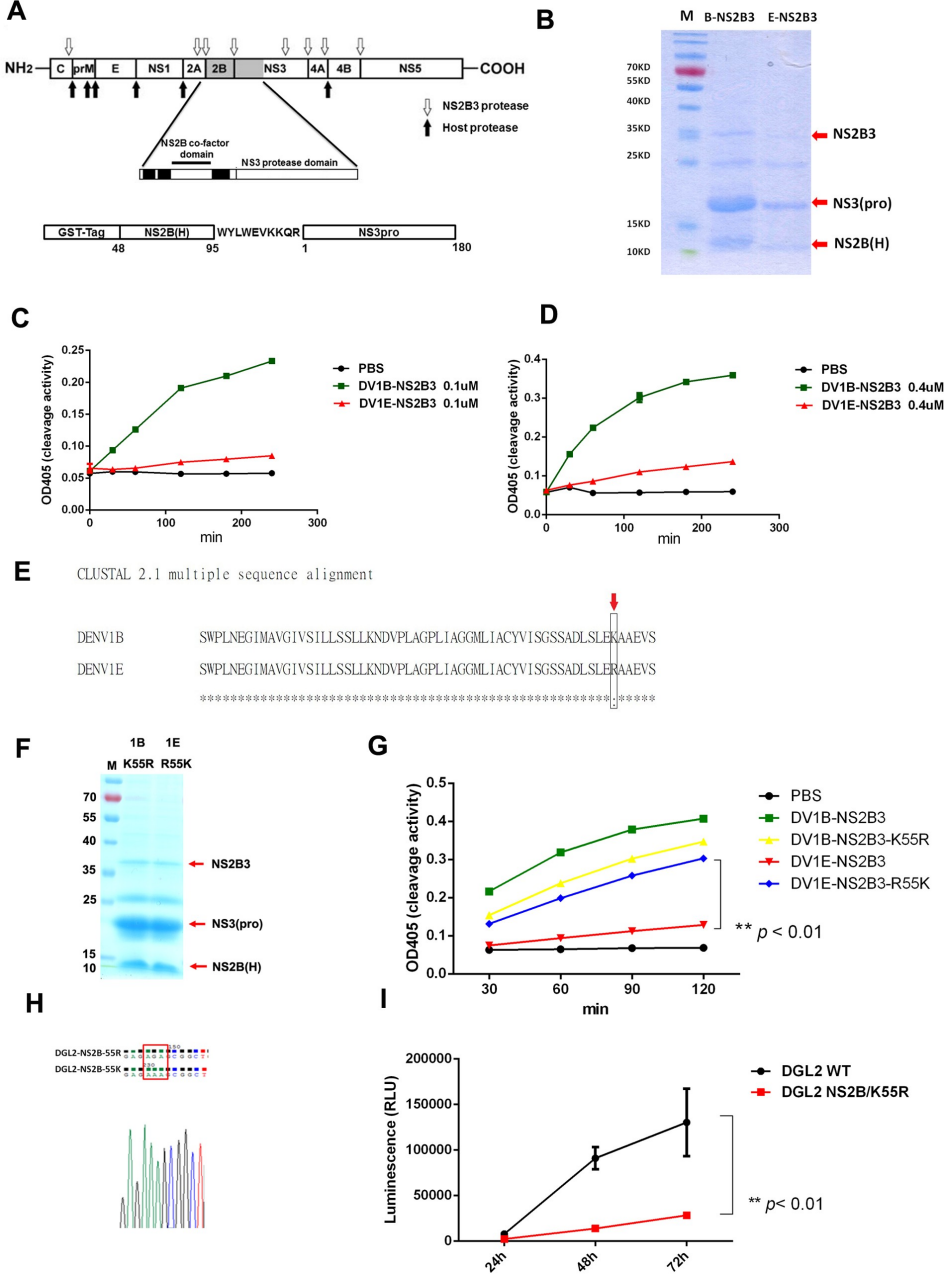
NS2B K55R is a virulent determination site for DENV1. (A) The schematic diagram of construction of mature NS2B3 protease. The NS2B co-factor domain (NS2B(H), 48–95 aa) and NS3 protease domain (NS3 1–180 aa) were cloned into a GST-fusion protein expression vector pGEX6p-2. (B) The recombinant protein purification of NS2B3pro from DENV1B and DENV1E. The auto-cleavage of NS2B3 was indicated by red arrows. (C-D) Enzymatic activity of NS2B3pro from DENV1B and DENV1E to a NS2B3-pNA substrate (Ac-EVKKQR-pNA). The OD value represents the cleavage efficiency of protease at indicated time points (min). The final enzyme concentrations were 0.1uM (C) and 0.4uM (D), respectively. (E) Protein sequence alignments of NS2B from DENV1B and E. (F) The expression the recombinant DENV1B NS2B3 K55R and 1E NS2B3 R55K mutant proteins. (G) The enzymatic activities of DENV1B, 1E WT and mutant NS2B3 proteins. (** *p*<0.01, ANOVA test) (H) The single point mutation of NS2B K55R in DGL2 replicon plasmid. (I) The replication efficiency of WT and NS2B K55R mutant replicons. (Results were shown as Mean±SEM, ** *p*<0.01, ANOVA test). The data shown are representative of at least 3 independent experiments.

The results above remind us that the amino acid variations may not only contribute to the difference in virus-host interaction, but also determine the replication ability of the virus itself. In DENV’s life cycle, NS2B forms a complex with NS3, and plays an important role in viral polyprotein procession. We then try to address whether amino acid changes in NS2B will directly influence viral protease function as well as viral replication. The mature forms of NS2B3 protease, which has a 48 amino acids NS2B co-factor domain (48–95 aa) and 180 amino acids NS3 protease domain, have been cloned and expressed in recombinant GST prokaryotic expression system (**Fig 5A and 5B**). The enzymatic assay suggested that NS2B3 protease from DENV1B showed higher substrate cleavage efficiency than NS2B3 from DENV1E (**Fig 5C and 5D**). Using site-directed single point mutation technology, we made a K55R mutation in NS2B3 of DENV1B, in which the 55^th^ amino acid Lys (K) was changed to Arg (R) (which is from DENV1E NS2B), as well as a R55K mutation to NS2B3 of DENV1E (**Fig 5E and 5F**). The enzymatic test showed that DENV1B NS2B3-K55R protein had lower enzymatic activity than wild type NS2B3 from DENV1B, while DENV1E NS2B3-R55K showed higher cleavage activity than wild type DENV1E NS2B3 (**Fig 5G**). We then made a NS2B K55R mutation to DENV1 replicon DGL2 (**Fig 5H**), and tested its replication efficiency. NS2B-K55R DGL2 replicon showed significantly lower replication efficiency than WT replicon (**Fig 5I**). These results suggested that NS2B K55 is a virulence determinant that important for DENV1 NS2B3 protease activity and viral replication. While, there are also several amino acid differences in the NS3 1–180 protease domain between DENV1B and DENV1E. Our preliminary data suggested that these mutations may also slightly influence the activity of NS2B3 activity. Further study need be performed to characterize other potential virulence determinants in NS3 protease domain.

## Discussion

Viruses are small infectious agents that replicates only inside the living cells of other organisms. The viral genome only encodes a limited number of proteins which are necessary for viral structure and replication. Instead, viruses use the machinery and metabolism of a host cell to complete their life cycles. At the same time, viral infections provoke an immune response that usually eliminates the infecting virus. To counteract the host defense mechanism, many viruses have evolved suppressor proteins to overcome the antiviral responses. So that, the virulence of a virus will be determined by two aspects: one is the ability of virus utilizing or antagonizing host responses, the other is the essential functions of those viral proteins.

A number of studies have reported single mutations in flavivirus protein influence the viral-host interactions, thereby determining the virulence of distinct virus. Yuan L *et al*. reported that S139N mutation in preM protein significantly increased the neurovirulence of Zika virus (ZIKV), and this could be the reason why ZIKV caused more microcephaly since the outbreak of 2010s[29]. Xia H *et al*. identified the A188V substitution in ZIKV NS1, which enhancing its IFN antagonizing activity [30]. The N124D and K128E mutations in DENV2 E protein reduced its heparin sulfate binding activity, and weakened the infectivity of mutated viruses[15]. In our current study, we also noticed that DENV1B NS proteins have stronger inhibitory ability against host IFN signaling. The A64V mutation in NS2A impaired the replication of DENV1, suggesting that A64 NS2A is a novel virulence determinant that may influence virus-host interaction.

At the same time, other studies suggested that variations in viral proteins may directly influence the viral protein functions. Some of DENV virulence determinants have been described, most of which locate at the E protein[14]. For example, the N67Q mutation in DENV2 E protein decreased virus growth, and N67 was identified as an important N-glycosylation site for this protein which is critical for viral assemble and budding[31]. Some substitutions in NS1, NS4B, and NS5 proteins were evidenced to increase viral replicative fitness in native mosquitoes[32]. In this study, we also identified two novel virulence determinants in the genomes of DENV1. The K55R substitution in NS2B results in an impaired protease activity of NS2B3, thereby compromised the viral replication efficiency. The 64^th^ amino acid of NS2A was also important for DENV1 replicon replication. The transmembrane topological structure of NS2A and NS2B[33–35], as well as the 3D crystal structure of NS2B3[36], were reported previously by several groups. By sequence alignment analysis, we found that 55^th^ amino acid residue of NS2B was located near the end of the first *β-*strand structure of the NS2B cofactor domain, which could be critical for stabilization of NS3 protease. Mutations made to the 63-65^th^ amino acids of NS2A displayed a lethal phenotype to DENV2 virus[35]. The 64^th^ amino acid residue was located in the top of the hinge area of the third transmembrane helix domain which may influence the topology of NS2A. Beside of this, molecular evolution analysis also suggested an A9G variation in Capsid protein between DENV1 B and DENV1 E (**S2 Table**). In the ViPR database, almost all of the Capsid proteins from DENV1 strains are A9, and only 52 strains which are G9. This suggests that the 9^th^ Alanine residue may be the dominant virulence loci. There are also a 20nt deletion in 3’UTR region of DENV1 D and E compared with DENV1 A-C, and this deletion may interfere with the stability of a SL1 loop in the 3’UTR, which is critical for the sfRNA generation[21]. Further study will be required to explore those potential virulence determinants for DENV1. We should also mention that even though we have confirmed that NS2A A64V and NS2B K55R mutations in replicons of DENV1B backbone have defect replication efficiencies than wild type replicon, the reverse mutations of these amino acids in a replicon with DENV1E backbone should also be important to support these findings. Further experiments will be performed to address this question.

Taken together, we compared the difference of replication efficiency and virulence of five DENV1 variants. We found that DENV1B is the most virulent virus, and DENV1E is the weakest. We further suggested that the 64^th^ amino acid of NS2A and 55^th^ amino acid of NS2B were potential virulence determinants of DENV1, which provided a theoretical basis for better understanding the molecular mechanisms of DENV virulence. It also provides new ideas for investigation of DENV protein function, pathogenic mechanism and novel attenuated vaccine.

## Materials and methods

### Ethics statements

The HUVEC (Human Umbilical Vascular Endothelium Cells) and PBMC (human Peripheral Blood Mononuclear Cells) were obtained from BeNa Culture Collection (Bejing, China). The projects using of human biological specimens were approved by an institutional review board (IRB) of Soochow University.

Animal experiments were conducted according to the Guide for the Care and Use of Medical Laboratory Animals (Ministry of Health, People’s Republic of China) and approved by the Animal Care and Use Committee as well as the Ethical Committee of Soochow University (No. SYSK-(S2012-0062)).

### Virus, cells and mice

Five different DENV1 viruses, isolated from the DENV outbreak of 2014 in Guangdong, were obtained from CDC of Guangdong province. The viruses were propagated in mosquito C6/36 cells (ATCC CRL-1660). 293T, Huh7.0 and Vero cells were obtained from ATCC (Manassas, USA) and grown in DMEM (Life Technologies, Grand Island, USA) supplemented with 10% FBS and antibiotics/antimycotics. HUVECs were grown in 1640 (Life Technologies) supplemented with 10% FBS and antibiotics/antimycotics. Mouse embryonic fibroblasts (MEFs) were prepared from the mouse embryo using standard protocols [37]. Cells were infected with DENV at a multiplicity of infection (MOI) of 0.5, unless otherwise stated.

BABL/C and C57BL/6J mice were obtained from Shanghai Laboratory Animal Center (Shanghai, China). IFNAR1^-/-^ mice (in a C57BL/6J background) were prepared by Institute of medical laboratory animal research, Chinese Academy of Medical Sciences (Beijing, China). All the animals were maintained in a biosafety level 2 animal facilities.

### RNA isolation and qRT-PCR

Total RNA from DENV infected cells were extracted using the total RNA kit I (OMEGA, USA) and reverse-transcribed using the PrimeScript Master Mix kit (TaKaRa, Japan). cDNAs were mixed with RT-PCR primers and SYBR Premix Ex Taq II (TaKaRa, Japan) and amplified for 40 cycles (95°C 15 s, 60°C 30 s, and 72°C 15s). The intracellular viral loads, in terms of transcript levels of the specific viral genes, were quantified through qRT-PCR and normalized to *β-actin* gene. The mRNA expression levels of human *IFNβ1*, *IFIT1*, and *Cig5* genes were also determined *via* qRT-PCR. (Oligo-primer sequences for qRT-PCR of this study were shown in **S1 Table**).

### Virus titration

The titers of DENV in cell-free supernatants or tissue extracts were determined with a median tissue culture infective dose (TCID50) assay and plaque assay according to protocols previously described [38,39], with slight modifications. Briefly, samples were serially diluted and inoculated into Vero cells in 96-well plates. After 5-day incubation, cells were fixed with 4% paraformaldehyde, stained with 10% crystal violet buffer, and examined for cytopathic effects (CPE) and plaque formation under a light microscope. The virus titer (TCID50/ml) was calculated using the Reed-Muench method. 1 TCID50/ml was equivalent to 0.69 *pfu*/ml[40].

### DENV replicon Gaussia luciferase reporter assay

DNA-based replicons (for DENV type 1) expressing secreted Gaussia luciferase (DGL2), were generously provided by Dr. Takayuki Hishiki (Kyoto University, Kyoto, Japan)[28]. The point mutations to the DGL2 replicon were obtained by using the QuickChange Site-directed Mutagenesis kit (Agilent Technologies, USA) according to manufacturer’s instructions.

For the Gaussia luciferase assay, 50 ng of DGL2 replicon plasmid was transfected into 293T cells in 96-well plates. Culture supernatants were collected at different time points and luciferase was measured using BioLux Gaussia Luciferase Assay Kit (New England Biolabs, UK) according to manufacturer’s instructions.

### DENV infection of newborn BABL/C mice and adult IFNAR1^-/-^ mice

Each 3- or 4-day-old BABL/C suckling mouse was inoculated intracerebrally with 100 PFU of DENV1 A-E respectively as previously described[41]. Animals were monitored for 21 days to evaluate the morbidity and mortality. The DENV replication levels in cerebrum at day 4 were measured by qRT-PCR method described above.

For infection of IFNAR1^-/-^ mice, 4–6 week-old IFNAR1^-/-^ mice were infected with 1×10^7^ PFU of DENV1B or DENV1E respectively by intraperitoneal injection. At day 3 post infection, mice were euthanized and the viral loads in whole blood cells and spleens were determined by qRT-PCR and plaque assays as described above.

### Plasmid construction, protein purification and western blot

The ORFs of NS2A, 2B, 4A and 4B from DENV1 A-E were subcloned into a eukaryotic expression vector pcDNA3.1A-His/Myc individually. The expressions of NS proteins were confirmed by western blot using anti-His Antibody (Sigma, USA). Prokaryotic expression plasmids for protease NS2B3 of DENV1 B and E (and NS2B3 mutants) were constructed as described previously[11,18,42]. Briefly, the coding region of NS2B enzyme co-factor domain (48–95 aa) and NS3 protease domain (1–180 aa) were amplified by nested PCR using the primers listed in the **S1 Table**, and cloned into the pGEX-6p2 bacterial expression vector. Then the pGEX-NS2B3 constructs were transformed into *Escherichia coli* strain BL21 (DE3) for protein expression. The GST-tagged recombinant proteins were induced with 0.1mM IPTG at 30°C for 4 h and purified using GST affinity agarose (GE Healthcare, Sweden). The GST tag was removed by Prescission Protease (Sigma, USA).

### Luciferase reporter assays

IFNβ- or ISRE-Luciferase reporter assay was performed as described previously[39,43]. Briefly, 293T cells were transfected with IFNβ- Luc (or ISRE-Luc) (Firefly luciferase, experimental reporter, 100 ng/well) and pRL-TK reporter (Renilla luciferase, internal control, 5 ng/well) plasmids (Clontech, USA), IFNβ activator RIG-I-N (the active caspase recruitment domain (CARD) containing form of RIG-I), together with individual NS proteins from DENV1 A-E or vector control. (For ISRE-Luc assay, cells were also treated with *IFNα* (1000 U/ml) for 6h instead of stimulation with RIG-I-N transfection.) 24 h post-transfection, cells were lysed and the luciferase activity was measured using a Dual Glow kit according to the manufacturer’s instructions (Promega, USA).

### NS2B3 enzymatic assay

Various concentration of purified recombinant NS2B3 from DENV1B, E and 1B K55R (or 1E R55K) mutants were incubated with DENV1 substrate Ac-EVKKQR-pNA [42](GL Biochem, Shanghai, China) at 37°C for indicated time courses, respectively. Enzymatic assay were carried out with the following buffers: 50 mM Tris-HCl, 10mM NaCl , 20% glycerin, 1mM CHAPS, pH 9.2. The substrate cleavage efficiencies were analyzed by measuring the OD value at 405nm as described before[42].

### Statistical analysis

Prism 7 software (GraphPad Software) was used for survival curves, charts and statistical analyses. The significance of results was analyzed using ANOVA followed by Tukey’s test for multiple comparisons, Student’s *t*-test (for comparisons between two groups) and Log-rank (Mantel-Cox) Test (for survival data), with a cutoff *P* value of 0.05.

## Supporting information

S1 Fig Phylogenetic tree of 128 DENV1 virus strains based on the sequences of viral genome. Sequences for additional 123 DENV1 strains were obtained from ViPR database (www.viprbrc.org). The sequence alignment was performed by Mafft 7.394 software (https://mafft.cbrc.jp/alignment/server). The whole genome phylogenetic tree was constructed by Neighbor-Joining method. The viruses belonging to genotype I, IV and V of DENV1 were indicated in the phylogenetic tree.
(TIF)

S1 Table Sequences of Oligo-primers used in this study. (DOCX)

S2 Table The summary of ancestral sequences variation sites in DENV1 A to E. The differences of ancestral protein sequences between group DENV1 B/C/A and DENV1 D/E were analyzed by MEGA7 software and listed in the table.
(DOCX)
