# Supplementary figures and images for: Virulence difference of five type I dengue viruses and the intrinsic molecular mechanism
Source: PLoS Negl Trop Dis. 2019 Mar 4;13(3):e0007202. doi: 10.1371/journal.pntd.0007202 (PMC6417740; doi:10.1371/journal.pntd.0007202)

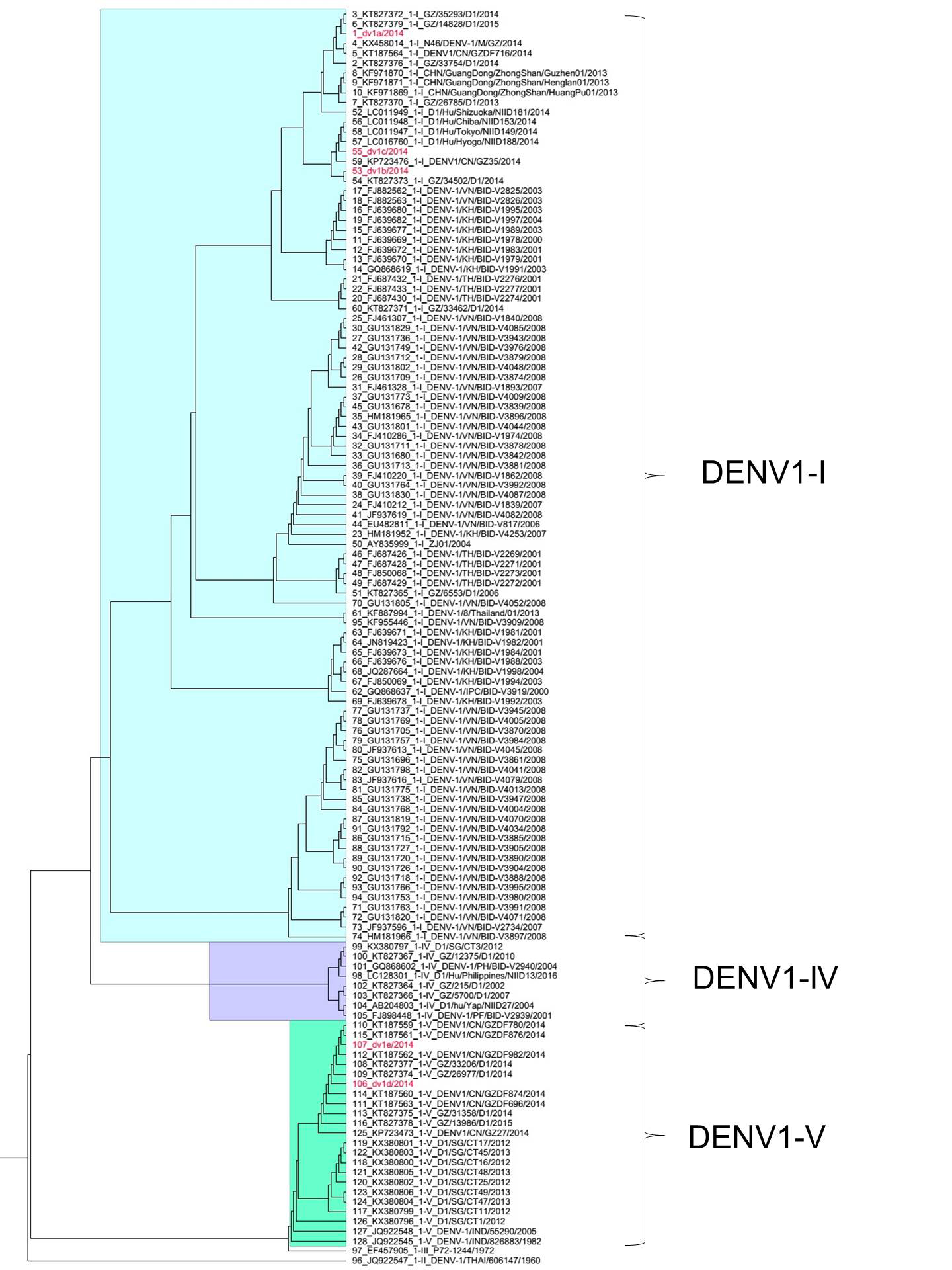

Supplement: S1 Fig — Sequences for additional 123 DENV1 strains were obtained from ViPR database (www.viprbrc.org). The sequence alignment was performed by Mafft 7.394 software (https://mafft.cbrc.jp/alignment/server). The whole genome phylogenetic tree was constructed by Neighbor-Joining method. The viruses belonging to genotype I, IV and V of DENV1 were indicated in the phylogenetic tree. (TIF) [file pntd.0007202.s001.tif]
