# Supplementary material for: Virulence difference of five type I dengue viruses and the intrinsic molecular mechanism
Source: PLoS Negl Trop Dis. 2019 Mar 4;13(3):e0007202. doi: 10.1371/journal.pntd.0007202 (PMC6417740; doi:10.1371/journal.pntd.0007202)
Supplement: S1 Table — (DOCX) [file pntd.0007202.s002.docx]

**Table S1. Sequences of Oligo-primers used in this study.**

| **Number** | **Sequence (5’-3’)** | **Purpose** |
| --- | --- | --- |
| P1 | AGAAGGAAATCACCGCCCTG | Forward primer for qRT-PCR of mosquito *β-actin* |
| P2 | GCTGGAAGGTGGATAGCGAG | Reverse primer for qRT-PCR of mosquito*β-actin* |
| P3 | TGACGTGGACATCCGCAAAG | Forward primer for qRT-PCR of human*β-actin* |
| P4 | CTGGAAGGTGGACAGCGAGG | Reverse primer for qRT-PCR of human*β-actin* |
| P5 | TAGAGAGCAGATCTCTGATGAA | Forward primer for qRT-PCR of DENV1 E gene |
| P6 | TGAGAATCTCTTCGCCAAC | Reverse primer for qRT-PCR of DENV1 E gene |
| P7 | CTTGGTACCGAGCTCGGATCCACCATGGATTACAAGGATGACGACGATAAGGGGTCAGGAGAAGTGGACAGTT | Forward primer for constructing plasmid pcDNA-DV1 2A |
| P8 | GAAGGGCCCTCTAGACTCGAGTTTCCTTCCCCAGATTTTGTT | Reverse primer for constructing plasmid pcDNA-DV1 2A |
| P9 | CTTGGTACCGAGCTCGGATCCACCATGGATTACAAGGATGACGACGATAAGAGCTGGCCTCTCAATGAAG | Forward primer for constructing plasmid pcDNA-DV1 2B |
| P10 | GAAGGGCCCTCTAGACTCGAGTCTCTGTTTCTTTTTCTGCCA | Reverse primer for constructing plasmid pcDNA-DV1 2B |
| P11 | CTTGGTACCGAGCTCGGATCCACCATGGATTACAAGGATGACGACGATAAGAGCGTCTCAGGTGACCTAA | Forward primer for constructing plasmid pcDNA-DV1 4A |
| P12 | GAAGGGCCCTCTAGACTCGAGGGCTGCCACTGTCAATATC | Reverse primer for constructing plasmid pcDNA-DV1 4A |
| P13 | CTTGGTACCGAGCTCGGATCCACCATGGACAATGAGATGGGATTACTGGAAACC | Forward primer for constructing plasmid pcDNA-DV1 4B |
| P14 | GAAGGGCCCTCTAGACTCGAGTCTCCTACCTCCTCCTAGAGATTT | Reverse primer for constructing plasmid pcDNA-DV1 4B |
| P15 | CCCTGGGATCCCCAGGAATTCCCGTCATATCTGGAAGCTCAGCC | Forward primer 1 for constructing plasmid pGEX-DV1B-NS2B3 |
| P16 | GCCAAAAGTACCAAGTTGCTTTAAGGAGAATGGTTAGC | Reverse primer 1 for constructing plasmid pGEX-DV1B-NS2B3 |
| P17 | AGCAACTTGGTACTTTTGGCAGAAAAAGAAA | Forward primer 2 for constructing plasmid pGEX-DV1B-NS2B3 |
| P18 | GTCACGATGCGGCCGCTCGAGATTATCTTTTCCTAAACACCTCATCTTCAA | Reverse primer 2 for constructing plasmid pGEX-DV1B-NS2B3 |
| P19 | CCCTGGGATCCCCAGGAATTCCCGTCATTTCCGGAAGCTCAGCC | Forward primer 1 for constructing plasmid pGEX-DV1E-NS2B3 |
| P20 | TACCAAGTTGCTTTGAGGAGTATAGTGAGCG | Reverse primer 1 for constructing plasmid pGEX-DV1E-NS2B3 |
| P21 | CTCCTCAAAGCAACTTGGTATTTTTGGCAGAAAAAGAAA | Forward primer 2 for constructing plasmid pGEX-DV1E-NS2B3 |
| P22 | GTCACGATGCGGCCGCTCGAGATTATCTTTTCTTAAACACCTCGTCCTCA | Reverse primer 2 for constructing plasmid pGEX-DV1E-NS2B3 |
| P23 | CCATCCTGTCGGAAACGTTGGCTCCAACC | Forward primer for point mutation of NS2A A64V |
| P24 | GGTTGGAGCCAACGTTTCCGACAGGATGG | Reverse primer for point mutation of NS2A A64V |
| P25 | GCATAGTGCAAGGATAACGTTGTTTTGATAAATGTCAAGGAC | Forward primer for point mutation of NS2A F159L |
| P26 | GTCCTTGACATTTATCAAAACAACGTTATCCTTGCACTATGC | Reverse primer for point mutation of NS2A F159L |
| P27 | CCTCAGCCGCTCTCTCTAGTGATAGATCGGCTG | Forward primer for point mutation of pGEX-DV1B-NS2B K55R |
| P28 | CAGCCGATCTATCACTAGAGAGAGCGGCTGAGG | Reverse primer for point mutation of pGEX-DV1B-NS2B K55R |
| P29 | CAGCCGATTTATCATTGGAGAAAGCGGCTGAGG | Forward primer for point mutation of pGEX-DV1E-NS2B R55K |
| P30 | CCTCAGCCGCTTTCTCCAATGATAAATCGGCTG | Reverse primer for point mutation of pGEX-DV1E-NS2B R55K |
| P31 | CCAACAAGTGTCTCCTCCAAAT | Forward primer for qRT-PCR of human *IFNβ1* |
| P32 | AATCTCCTCAGGGATGTCAAAGT | Reverse primer for qRT-PCR of human *IFNβ1* |
| P33 | TGACTCTTTTGCCTCTTTCTTCTAA | Forward primer for qRT-PCR of human *IFIT1* |
| P34 | TTCTTGGGGT GCTCTGTGG | Reverse primer for qRT-PCR of human *IFIT1* |
| P35 | TGGGTGCTTACACCTGCTG | Forward primer for qRT-PCR of human *Cig5* |
| P36 | GAAGTGATAGTTGACGCTGGTT | Reverse primer for qRT-PCR of human *Cig5* |
