# Supplementary material for: Virulence difference of five type I dengue viruses and the intrinsic molecular mechanism
Source: PLoS Negl Trop Dis. 2019 Mar 4;13(3):e0007202. doi: 10.1371/journal.pntd.0007202 (PMC6417740; doi:10.1371/journal.pntd.0007202)
Supplement: S2 Table — The differences of ancestral protein sequences between group DENV1 B/C/A and DENV1 D/E were analyzed by MEGA7 software and listed in the table. (DOCX) [file pntd.0007202.s003.docx]

| **Protein（aa positions）** | **variation sites** | **DV1/**  **ABC** | **DV1/DE** | **Protein（aa positions）** | **variation sites** | **DV1-ABC** | **DV1-DE** |
| --- | --- | --- | --- | --- | --- | --- | --- |
| capsid（5-114） | 9 | A | G |  | 1327 | L | F |
|  | 26 | V | S |  | 1338 | A | T |
|  | 46 | L | M | NS2B(1349-1475) | 1401 | K | R |
|  | 70 | G | S |  | 1458 | L | M |
|  | 90 | N | S | Peptidase S7, Flavivirus NS3 serine protease  (1493-1642) | 1519 | E | D |
|  | 109 | M | L |  | 1520 | N | G |
| Flavi_M(207-280) | 232 | K | R |  | 1561 | L | F |
|  | 236 | R | K |  | 1588 | A | T |
| Flavi_glycoprot(282-576) | 288 | S | N | Flavivirus DEAD domain(1661-1806) | 1661 | R | K |
|  | 317 | D | N | DEAD-like helicases superfamily  (1665-1827) | 1826 | E | D |
|  | 394 | I | L |  | 1928 | V | A |
|  | 435 | S | T |  | 1942 | H | Q |
|  | 441 | T | I |  | 2114 | Q | R |
|  | 451 | T | S | NS4B(2245-2486) | 2264 | A | V |
| Flavi_glycop_C  (578-673) | 577 | M | V |  | 2265 | V | A |
|  | 604 | I | V |  | 2272 | A | T |
|  | 617 | F | I |  | 2276 | V | I |
|  | 649 | A | T | FtsJ-like methyltransferase  (2548-2714) | 2617 | L | I |
| flavi_E_stem  (679-775) | 719 | I | V |  | 2624 | K | R |
|  | 741 | V | I |  | 2629 | I | T |
|  | 764 | L | M | NS5(2743-3386) | 2779 | H | N |
| NS1(777-1130) | 868 | V | A |  | 2819 | K | R |
|  | 869 | A | N |  | 2858 | K | R |
|  | 873 | A | L |  | 2859 | A | P |
|  | 887 | Y | - |  | 2872 | R | K |
|  | 904 | V | T |  | 3059 | K | T |
|  | 915 | N | D |  | 3060 | A | S |
|  | 938 | I | V |  | 3079 | N | S |
|  | 954 | V | M |  | 3110 | A | V |
|  | 1003 | K | R |  | 3129 | T | N |
|  | 1054 | D | N |  | 3130 | P | S |
|  | 1123 | K | R |  | 3143 | Y | H |
| NS2A(1138-1320) | 1143 | I | V |  | 3163 | I | T |
|  | 1192 | A | V |  | 3323 | Y | V |
|  | 1261 | I | M |  |  |  |  |
|  | 1275 | A | T |  |  |  |  |
|  | 1287 | F | L |  |  |  |  |
|  | 1290 | H | D |  |  |  |  |

Supplementary Table S2. **The summary of ancestral sequences variation sites in DENV1 A to E.**
